# Combined application of organic and microbial amendments reshapes soil microbiome diversity and improves soybean growth performance

**DOI:** 10.3389/fmicb.2026.1939259

**Published:** 2026-09-18

**Authors:** Xuanqi Zhang, Wenjie Dou, Qinqin Cao, Jing Guo, Yifan Yin, Zhiwei Zhu, Mengke Zeng, Jiale Liu, Dejun Han, Xiaoyu Qiang

**Affiliations:** 1 State Key Laboratory of Crop Stress Resistance and High-Efficiency Production, College of Agronomy, Northwest A&F University, Yangling, Shaanxi, China; 2 State Key Laboratory of Crop Stress Resistance and High-Efficiency Production, College of Plant Protection, Northwest A&F University, Yangling, Shaanxi, China; 3 Agricultural College, Inner Mongolia Minzu University, Tongliao, Inner Mongolia, China

**Keywords:** community composition, microbial diversity, rhizospheric microbiome, soil amendment, soybean

## Abstract

Agricultural practices, including organic amendments and microbial inoculants, strongly influence soil microbial communities, which play a crucial role in maintaining soil function and structure. To identify an effective strategy for improving soil microbiome diversity and crop production, we assessed and compared the soil microbial community in fields treated with cattle manure amendment (MA), microbial inoculant (MI), and a mixture of cattle manure and microbial inoculant (MM), while an untreated field served as the control. We then evaluated treatment effects on soybean production. Microbial diversity analyses showed that MM treatment was associated with increased bacterial diversity in bulk soil, whereas a pronounced enhancement of fungal diversity was observed in the rhizosphere. Community composition analyses indicated the elevation of relative abundances of Acidobacteriota and Chloroflexi in bulk soil upon either treatment. Notably, the Proteobacteria, including the rhizobial genus *Ensifer*, was significantly enriched in the rhizosphere upon MM treatment, while the relative abundance of Ascomycota was increased in both bulk and rhizospheric soil upon MA treatment. To co-relate with these soil microbiome features, the MM treatment was shown to have the most significant effect on the soybean growth promotion, including the notably higher aboveground and underground fresh weight, plant height, accompanied with markedly more root nodules, than either MI or MA treatment. Collectively, our results suggest that the combined application of organic and microbial amendments effectively reshapes the soil microbiome by enhancing microbial diversity and enriching nitrogen-fixing bacteria in the rhizosphere, thereby promoting the nitrogen-fixing potential and productivity of soybean.

## Introduction

Soil microorganisms play central roles in maintaining soil ecosystem functions and stability (Fierer, 2017). As key drivers of nutrient cycling and biochemical transformations, they profoundly influence plant growth, productivity, and health (Yang et al., 2023). Through complex and tightly connected interaction networks, soil microbiomes enhance both structural stability and functional resilience of soils (Fan et al., 2017). Advances in high-throughput sequencing have greatly improved our ability to characterize these communities and infer their functional contributions to plant performance (Pang et al., 2021). Combining sequencing with network-based analyses thus offers a powerful framework to quantify microbial compositional shifts and interaction patterns across soil habitats and management regimes (Wang et al., 2021).

Given their central role in soil functioning, soil microbial communities are strongly shaped by agricultural inputs and management interventions (Fox et al., 2022). Beyond conventional fertilization regimes, increasing attention has been paid to organic amendments and microbial inoculants as sustainable strategies to improve soil fertility, suppress soil-borne diseases, and enhance crop productivity (Tao et al., 2020; Liu et al., 2021). Organic amendments such as composted cattle manure can increase soil organic matter availability, stimulate microbial activity, and enhance soil suppressiveness against soil-borne pathogens (Das et al., 2017). Microbial inoculants have the potential to promote plant growth and modulate rhizosphere microbial communities through mechanisms such as nutrient mobilization, phytohormone production, and suppression of soil-borne pathogens (Souza et al., 2015). However, their efficacy under field conditions is often influenced by soil properties (Azarbad and Junker, 2024), environmental conditions and interactions with native microbial communities (Overall et al., 2025). Moreover, formulating inoculants with organic carriers such as composted manure can improve inoculant survival and field performance, suggesting that combined manure-inoculant strategies may offer synergistic benefits in agroecosystems (Zhou et al., 2024; Shilpa and Brahmaprakash, 2016). Therefore, elucidating how different amendment strategies regulate microbial community composition and interaction networks across distinct soil compartments is critical for developing effective and sustainable field management practices.

Numerous studies have shown that organic amendments and microbial inoculants are key drivers of soil microbial community structure and function, contributing to soil health improvements (Elnahal et al., 2022; Beillouin et al., 2022). Meta-analyses further indicate that microbial inoculation can increase microbial biomass carbon and nitrogen, modulate bacterial composition, and enhance network stability, although these effects are context-dependent, influenced by soil nutrient status, environmental stresses, and the use of native versus non-native inoculants (Li et al., 2024). Despite these insights, the interplay between organic amendments, microbial inoculants, and soil microbial communities remains poorly understood, particularly under rotational cropping systems (Christensen et al., 2026). Soybean (*Glycine max* L.) is an economically important leguminous crop worldwide (Poole et al., 2018), and its growth and productivity are closely associated with rhizosphere microbial interactions, particularly symbiotic relationships with rhizobia that contribute to biological nitrogen fixation (Philippot et al., 2013). Wheat (*Triticum aestivum* L.) is one of the world’s major cereal crops and is widely cultivated in diverse agricultural systems, contributing substantially to global food production (Shiferaw et al., 2013). Wheat-soybean rotation brings together two agronomically important crops with distinct crop identities and resource-use characteristics and is widely adopted in many agricultural regions (Qin et al., 2015). Compared with continuous monocropping, wheat-soybean rotation has been reported to diversify seasonal resource inputs (Tiemann et al., 2015), improve soil nutrient cycling (McDaniel et al., 2014), and influence microbial community assembly through changes in crop identity and plant-derived carbon inputs (Vandenkoornhuyse et al., 2015). These differences in crop identity and resource inputs may shape soil ecological conditions and influence crop-microbe interactions and microbial community assembly (Cerasuolo et al., 2026). Therefore, wheat-soybean rotation represents an important field context for investigating how organic amendments and microbial inoculants reshape soybean-associated microbial communities and affect plant performance (Qin et al., 2015). While each practice can independently improve soil fertility and plant performance, their combined effects may be non-additive due to complex plant-microbe-soil feedbacks (Ma et al., 2018). Furthermore, few studies have simultaneously investigated how these amendments influence bacterial and fungal communities across distinct soil compartments (Guo et al., 2025). Addressing these knowledge gaps is critical to elucidating the synergistic potential of integrated amendment strategies for sustainable agroecosystem management.

Root-derived inputs, including exudates, secretions, and mucilage, strongly influence microbial-mediated transformations of carbon, nitrogen, and phosphorus, leading to distinct chemical and microbial profiles in the rhizosphere (Mueller et al., 2024). As the primary source of microbial inoculum to the rhizosphere, the composition of bulk soil communities plays a pivotal role in shaping rhizosphere microbial assembly (Zhong et al., 2022). The rhizosphere, directly influenced by root exudates and associated plant-driven processes (Li et al., 2021), often exhibits greater resistance to environmental perturbations, while bulk soil serves as the primary microbial reservoir from which rhizosphere communities assemble (Tong et al., 2025; Zhou et al., 2020). Therefore, simultaneous analysis of rhizospheric and bulk soil compartments is critical to comprehensively characterize microbial responses to field management practices (Ling et al., 2022), enabling a mechanistic understanding of how soil microbial communities regulate nutrient dynamics and crop growth.

In this study, we evaluated the effects of three field management strategies involving different amendment practices on soil and plant responses under field conditions. We sampled both rhizospheric and adjacent bulk soil at the soybean pod-filling (R5) stage, a critical period when root activity and rhizodeposition peak and rhizosphere microbial communities exhibit high abundance, stability, and functional specificity, while the plant undergoes key reproductive development (Wang et al., 2024). To assess soil microbiome responses, we performed amplicon sequencing targeting bacterial 16S rRNA genes and fungal internal transcribed spacer (ITS) regions, followed by analyses of microbial diversity, community composition, heatmap visualization, and differential taxa using linear discriminant analysis effect size (LEfSe). These approaches, together with phenotypic measurements and correlation analysis, enabled a systematic investigation of how different soil amendments influence microbial community structure and their associations with plant performance in both rhizospheric and bulk soil.

## Materials and methods

### Sample collection and experimental design

Field experiments were conducted in a wheat–soybean rotation field at the Doukou Experimental Station of Northwest A&F University, Jingyang County, Shaanxi Province, China (34°36′N, 108°52′E). The soil is classified as a clay loam (USDA), corresponding to a Eumorthic Anthrosol under the WRB classification (Zhao et al., 2019). Based on previously reported measurements from the same experimental station, the baseline soil chemical properties of the 0–20 cm soil layer were as follows: soil organic matter was 16.99 g kg^−1^, total nitrogen was 1.15 g kg^−1^, total phosphorus was 0.55 g kg^−1^, and available potassium was 286.00 mg kg^−1^ (Wen et al., 2024). Four treatments were established: untreated control (CK), cattle manure amendment (MA), commercial microbial inoculant treatment composed of *Bacillus* strains (MI), and a combined cattle manure and microbial inoculant treatment (MM). For the MA and MM treatments, cattle manure was applied at a rate of 15 t ha^−1^ before soybean sowing. For the MI and MM treatments, the commercial microbial inoculant was applied at a rate of 1 kg ha^−1^ before sowing. For the MM treatment, the microbial inoculant was thoroughly mixed with cattle manure before field application. The application rates of cattle manure and microbial inoculant in the MM treatment were identical to those used in the corresponding single-amendment treatments. Treatments were arranged in a randomized complete block design with five replicates per treatment. Each plot measured 10 × 10 m (100 m^2^). Field management (including irrigation and weed control) followed local farmers’ conventional practices and was applied uniformly across all plots.

Plant growth measurements and soil samples were collected at the soybean pod-filling stage (R5), a period characterized by active plant growth, substantial root activity, and rhizodeposition (Zhong et al., 2019). This stage was selected to evaluate the effects of microbial inoculation on soybean growth and soil microbial communities. Within each plot, a five-point sampling pattern was adopted. Samples were collected from the four corners and the center of each plot. At each of the five points, three soybean plants were excavated and combined to form one composite sample per plot. Sampling positions for each campaign were shifted relative to prior samplings to avoid resampling the same plants. For each sampled plant, the whole plant was carefully excavated and loose soil was gently shaken off in the field. Root segments of 5–15 cm length were then cut and placed into sterile 50 mL centrifuge tubes for rhizospheric processing (Lu et al., 2018). Bulk soil was collected from the adjacent root-free zone approximately 5–10 cm away from the root system and from areas between rows to avoid rhizosphere influence (Liu et al., 2022). All samples were placed in an ice box immediately after collection, transported to the laboratory, and then stored at −80 °C until DNA extraction.

Rhizospheric soil was defined as soil tightly adhering to roots (Barillot et al., 2012). Each root sample was placed in 25 mL phosphate-buffered saline (PBS; 137 mM NaCl, 2.7 mM KCl, 10 mM Na₂HPO₄·12H₂O, 2 mM KH₂PO₄), sonicated at 40 Hz for 1 min, and shaken to dislodge rhizospheric soil. The suspension was transferred to a new sterile tube and centrifuged at 9,000 rpm for 5 min to pellet rhizospheric soil (Wang et al., 2020; Edwards et al., 2015). Bulk soil was gently sieved through a 2 mm mesh to remove stones and roots, while rhizospheric pellets were collected directly. Both soil types were stored at −80 °C until DNA extraction.

### DNA extraction and PCR amplification

Total genomic DNA was extracted from soil samples using the FastDNA® Spin Kit for Soil (MP Biomedicals, USA) following the manufacturer’s instructions. DNA concentration and purity were assessed using a NanoDrop 2000 spectrophotometer (Thermo Fisher Scientific, USA). For bacterial community analysis, the V3-V4 region of the 16S rRNA gene was amplified using the primer pair ACTCCTACGGGAGGCAGCA (forward) and GGACTACHVGGGTWTCTAAT (reverse), with approximately 50 ng of DNA per reaction.

For fungal community analysis, the ITS1–2 region was amplified using the primers CTTGGTCATTTAGAGGAAGTAA (forward) and GCTGCGTTCTTCATCGATGC (reverse). PCR amplification programs for both bacterial and fungal targets are provided in Supplementary Tables S1, S2. All PCR reactions were performed in triplicate and pooled to minimize amplification bias. The amplicons were purified, quantified, and sequenced on the Illumina MiSeq platform (2 × 300 bp paired-end) at Meggene Co., Ltd. (Shenzhen, China).

### Bioinformatic analysis

Raw paired-end reads were imported into QIIME2 (v2024.2) and demultiplexed by sample-specific barcodes. Primer sequences and adapters were trimmed using the *q2-cutadapt* plugin. Quality filtering, denoising, paired-end merging, and chimera removal were conducted with the DADA2 algorithm (*q2-dada2*) to infer amplicon sequence variants (ASVs) (Callahan et al., 2016). Taxonomic assignment of bacterial and fungal ASVs was conducted using the SILVA (v138) and UNITE (v8.0) reference databases (Abarenkov et al., 2024; Quast et al., 2013), respectively, via a pre-trained naïve Bayes classifier. Non-target sequences (chloroplast, mitochondria, and non-bacterial or non-fungal ASVs) and singletons were removed prior to downstream analyses. Alpha diversity (Chao1) was calculated using QIIME2 and the *phyloseq* package in R (v4.3.2) (Zhang et al., 2024). All samples were rarefied to the minimum sequencing depth to standardize sampling effort. Statistical differences among treatments were tested using one-way ANOVA or Kruskal-Wallis tests followed by Benjamini-Hochberg (FDR)-corrected *post-hoc* comparisons (McMurdie and Holmes, 2013). Beta diversity was evaluated using Bray–Curtis dissimilarity and weighted UniFrac distances, and visualized via principal coordinate analysis (PCoA). Differences in community composition among treatments were further assessed by PERMANOVA (*adonis2, vegan package*) based on 999 permutations. Taxonomic composition was summarized from phylum to genus levels and visualized as stacked bar plots (McMurdie and Holmes, 2013). Differentially abundant taxa among treatments were identified using the linear discriminant analysis effect size (LEfSe) method with an LDA score threshold of 3.5 and *α* = 0.05. Correlation between genus-level relative abundance and nodule number was evaluated using Spearman’s rank correlation in R (cor.test), and visualized with *ggplot2* and *ggpubr*. All statistical analyses and plotting were performed in R (v4.3.2) with the packages *phyloseq*, *vegan*, *ggplot2*, and *ggClusterNet*.

## Results

### Bacterial community structural analyses in bulk and rhizospheric soil upon different treatments

To evaluate the effects of different field treatments (i.e., MI, MA and MM) on soil bacterial communities, 40 soil samples were harvested for 16 s rRNA amplicon sequencing and 2,313,209 high-quality, nonchimeric sequences were obtained in total, with a median of 59,180 sequences per sample (range: 39,126–89,427). Sequencing data were rarefied to the minimum sequencing depth to ensure comparability across samples, resulting in the identification of 43,025 bacterial ASVs. Based on these normalized datasets, we assessed bacterial alpha diversity using the Chao1 index, which primarily reflects community richness. In bulk soil, the MA and MI treatments showed slightly lower Chao1 richness compared with the control field, whereas the MM treatment exhibited the highest Chao1 index, which is significantly higher than that of the MA treatment (Figure 1A; *p* < 0.05). In contrast, no significant differences in Chao1 richness were observed among treatments in rhizospheric soil (Figure 1B).

**Figure 1:**
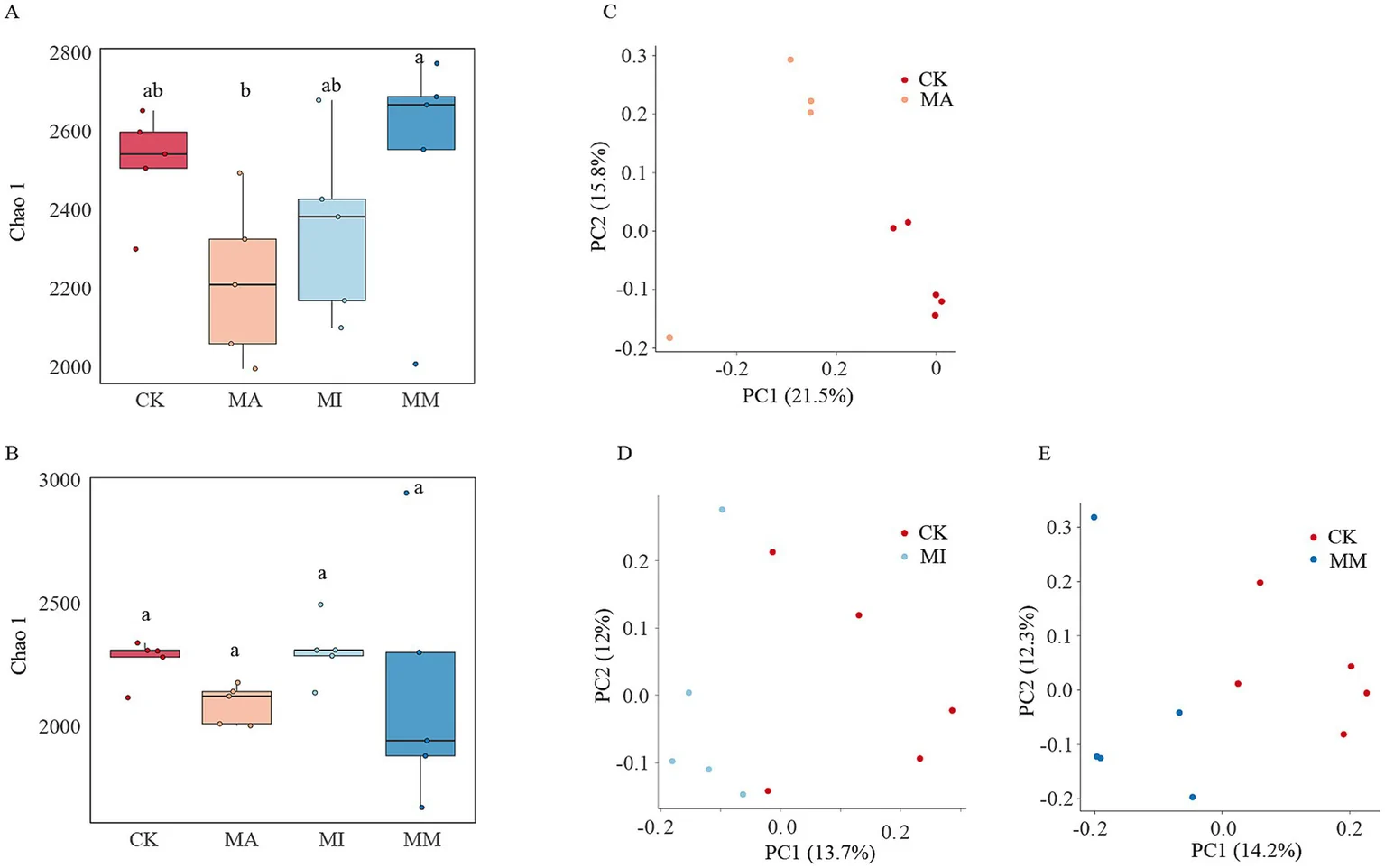
Alpha diversity and beta diversity of bacterial communities under different field treatments. **(A,B)** Chao1 α-diversity of bacterial communities in bulk soil **(A)** and rhizospheric soil **(B)**. Different lowercase letters indicate significant differences among treatments based on one-way ANOVA followed by LSD *post-hoc* tests (*p* < 0.05). **(C–E)** Principal coordinate analysis (PCoA) of rhizospheric bacterial community composition based on weighted UniFrac distances, showing pairwise comparisons between each treatment and the control (CK): MA vs. CK [**(C)**, PC1 = 21.5%, PC2 = 15.8%], MI vs. CK [**(D),** PC1 = 13.7%, PC2 = 12.0%], and MM vs. CK [**(E),** PC1 = 14.2%, PC2 = 12.3%]. Treatments include the untreated control (CK), cattle manure amendment (MA), commercial microbial inoculant (MI), and the combined manure-based inoculant treatment (MM).

Thereafter, bacterial *β*-diversity was examined on base of weighted UniFrac distances. The principal coordinate analysis (PCoA) of bulk soil bacterial communities revealed a clear separation between the MA and CK treatments, whereas communities under the MI and MM treatments largely overlapped with CK (Supplementary Figure S1). Notably, in rhizospheric soil, PCoA plots showed a pronounced separation of the MA treatment from CK, while the MM treatment exhibited a moderate shift and the MI treatment showed a slight separation relative to CK (Figures 1C–E). Collectively, these results indicate that soil bacterial communities were influenced, to varying extents, by the MA, MI, and MM treatments, with effects being more pronounced in the rhizosphere than in bulk soil.

Further analyses on bacterial community composition showed that, in bulk soil, Acidobacteriota and Proteobacteria were the dominant phyla, followed by Bacteroidota, Gemmatimonadota, Actinobacteriota, Chloroflexi, Myxococcota, Verrucomicrobiota, and Firmicutes (Figure 2A). Relative to CK, the MM, MA, and MI treatments resulted in higher relative abundances of Acidobacteriota, accompanied by variable shifts in several other bacterial phyla, including Gemmatimonadota, Chloroflexi. Consistently, the phylum-level heatmap further revealed distinct relative abundance patterns of dominant taxa across treatments, with clear compartment-specific differentiation between bulk and rhizospheric soils, with Gemmatimonadota, Chloroflexi, and Nitrospirota being preferentially enriched in bulk soil across all treatments (CK, MA, MI, and MM) (Figure 2D). At the genus level, no significant differences in overall community composition were detected among treatments (Supplementary Figure S2). In rhizospheric soil, the MM treatment enriched particularly Proteobacteria, whereas MA increased more abundance of Acidobacteriota, followed by Bacteroidota, Actinobacteriota, Verrucomicrobiota, Gemmatimonadota, Chloroflexi, Firmicutes, and Myxococcota, which were dominant across treatments (Figure 2B). Notably, at the genus level, the MM treatment significantly enriched *Ensifer* (Figure 2C), a member of the *Rhizobiales* commonly associated with root symbioses and nitrogen cycling, whereas the MA treatment was characterized by a marked increase in *Vicinamibacteraceae* and *Bacillus.* Conversely, *Sphingomonas* showed consistent declines across the amended treatments compared with CK. Taken together, these results suggest that the MM treatment might contribute to recruit root-associated and potentially nitrogen fixing taxa.

**Figure 2:**
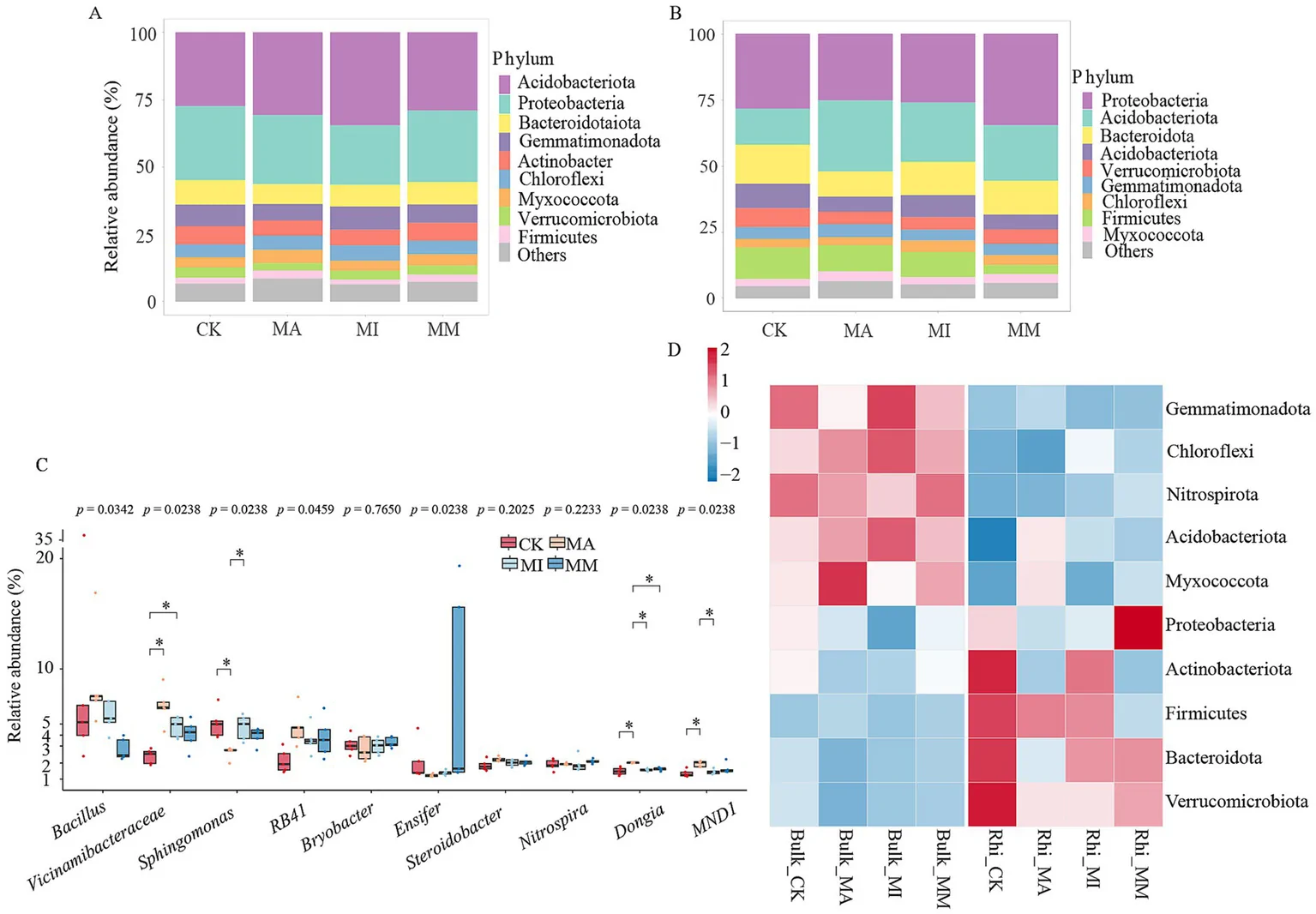
Relative abundance and community composition of bacterial taxa in bulk and rhizospheric soils under different field treatments. **(A,B)** Relative abundances of dominant bacterial phyla in bulk soil **(A)** and rhizospheric soil **(B)**, respectively. Different colors indicate different bacterial phyla. **(C)** Relative abundances of the top 10 most abundant bacterial genera in rhizospheric soil across treatments. **(D)** Phylum-level composition and clustering of bacterial communities in bulk and rhizospheric soils under different treatments.

### Fungal community structural analyses in bulk and rhizospheric soil upon different treatments

In total, 3,001,746 high-quality, nonchimeric fungal sequences were obtained from 40 samples, with a median of 55,754 sequences per sample (range: 41,377–254,825). Sequencing data were rarefied to the minimum sequencing depth to ensure comparability across samples, resulting in the identification of 4,638 fungal ASVs. We next examined fungal communities in both bulk and rhizospheric soils. In bulk soil, the Chao1 index showed slightly higher richness in the MI and MA treatments compared with CK, although these differences were not statistically significant (Supplementary Figure S3A). In rhizospheric soil, Chao1 richness was highest under MM and was significantly greater than in CK (Supplementary Figure S3B; *p* < 0.05), indicating that MM was associated with increased fungal richness in the rhizosphere. *β*-diversity analyses based on weighted UniFrac indicated fungal communities in bulk soil upon MA treatment showed a slight separation from the other treatments, whereas no clear separation was observed among MI, MM, and CK (Supplementary Figure S3C). In rhizospheric soil, *β*-diversity analysis did not reveal any evident separation of fungal community composition among treatments (Supplementary Figure S3D).

Nevertheless, fungal community composition in bulk soil exhibited treatment-dependent shifts (Figure 3A). All three treatments increased the relative abundance of Ascomycota compared with the control, with the MA treatment showing the highest proportion. In contrast, Basidiomycota displayed varying levels of decline across the treatments. And the fungal phyla Chytridiomycota reached its highest relative abundance under MI; Mortierellomycota was mostly enriched in MA, and Rozellomycota peaked in MM (Figure 3A). Consistently, the phylum-level heatmap further highlighted these patterns, with MA enriched in Mortierellomycota and Monoblepharomycota, while MI was characterized by higher relative abundances of Zoopagomycota, and both MI and MM showing enrichment of Chytridiomycota. (Figure 3D). Given the limited taxonomic resolution at the genus level for a substantial proportion of fungal ASVs, we further compared dominant taxa at the family level (Supplementary Figure S4). The MA treatment resulted in increased abundances of Chaetomiaceae and Lasiosphaeriaceae, whereas the MM treatment was associated with enrichment of Nectriaceae. In rhizospheric soil, Ascomycota and Basidiomycota dominated the fungal community and reached their highest relative abundances under the MA treatment (Figure 3B), indicating an overall enrichment of these two phyla in the rhizosphere following manure amendment. In contrast, Rozellomycota peaked under the MM treatment. Several other phyla, including Glomeromycota, Mortierellomycota, Chytridiomycota, Zoopagomycota, and Blastocladiomycota, also exhibited treatment-associated responses but remained consistently low in relative abundance (<1%) across samples. Notably, the relative abundances of these minor phyla were generally lower in rhizospheric soil than in bulk soil, suggesting additional selective filtering during the transition from bulk soil to the root-associated niche. Consistently, the phylum-level heatmap further revealed a pronounced enrichment of Ascomycota and Blastocladiomycota under MA, whereas Rozellomycota was preferentially enriched under MM (Figure 3D). At the family level, both MA and MI treatments increased the relative abundance of Chaetomiaceae, whereas Aspergillaceae was specifically enriched under MA (Figure 3C). In contrast, the MM treatment was associated with higher relative abundances of Ceratobasidiaceae, Bolbitiaceae, and Glomeraceae. Together, these results indicate that soil amendments induced markedly greater overall shifts in microbial community structure in rhizospheric soil than in bulk soil.

**Figure 3:**
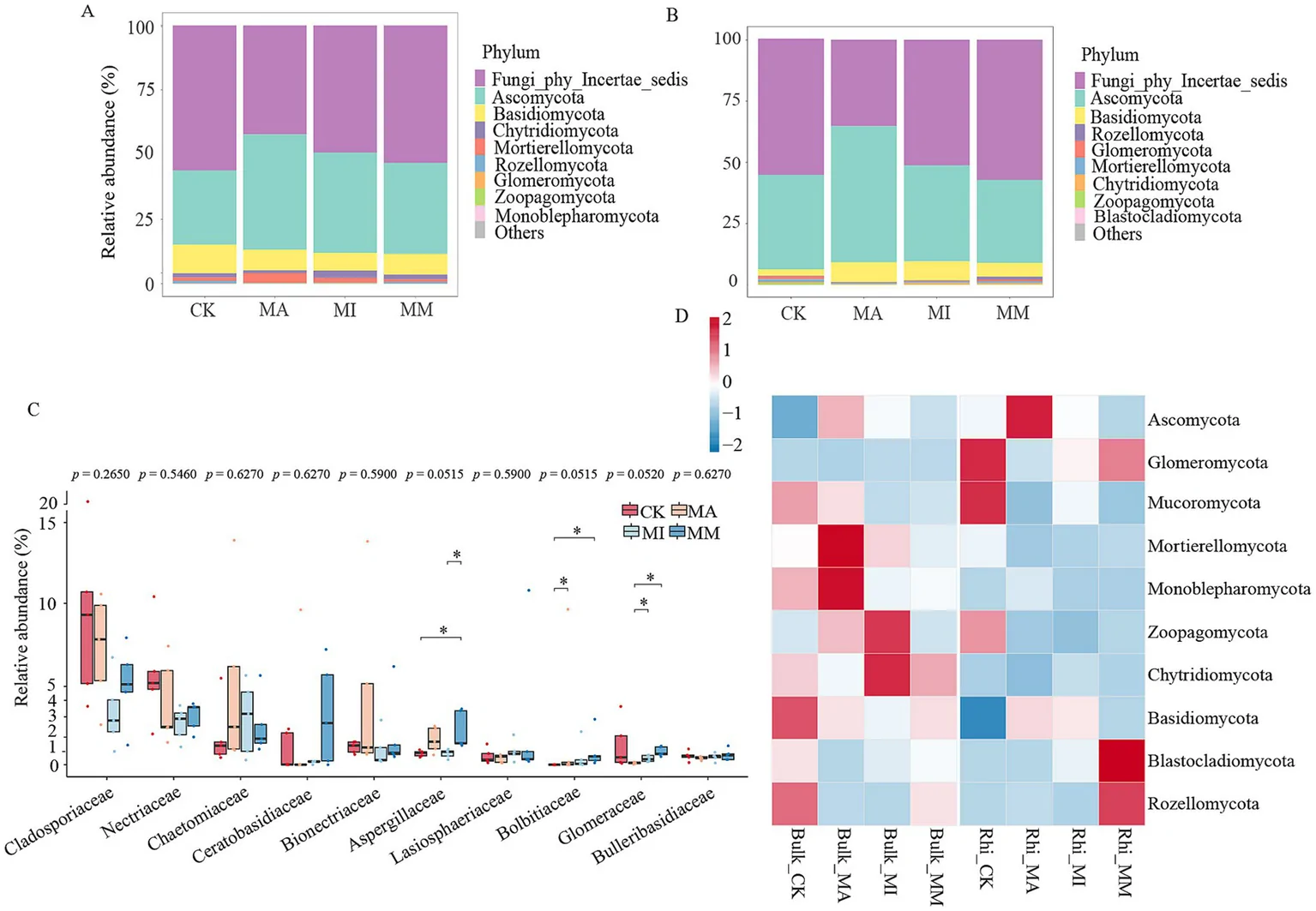
Relative abundance and community composition of fungal taxa in bulk and rhizospheric soils under different field treatments. **(A,B)** Relative abundances of dominant fungal phyla in bulk soil **(A)** and rhizospheric soil **(B)**, respectively. **(C)** Relative abundances of the top 10 most abundant fungal families in rhizospheric soil across treatments. **(D)** Phylum-level composition and clustering of fungal communities in bulk and rhizospheric soils under different treatments.

### Treatment-specific microbial taxa in the rhizosphere

To further resolve the taxa underlying the observed compositional shifts in rhizospheric communities, LEfSe analysis was performed at the genus level using an LDA score threshold of 3.5 and a significance level of *p* < 0.05. In the MA treatment, several bacterial genera, including *Vicinamibacteraceae*, *Dongia*, *Chryseolinea*, *Rokubacteriales*, and *Acidibacter*, were significantly enriched. In the MI treatment, *Pseudoxanthomonas* and *Chitinophaga* were identified as indicator bacterial genera. Notably, the MM treatment selectively enriched *Ensifer*, which exhibited the highest LDA score among all discriminative bacterial taxa, along with *Terrimonas*, suggesting that the genus represent key bacterial indicators associated with the MM treatment (Figure 4A). For fungal communities, LEfSe analysis revealed that *Penicillium* was significantly enriched in the MA treatment, whereas *Conocybe* and *Aspergillus* were preferentially associated with the MM treatment (Figure 4B).

**Figure 4:**
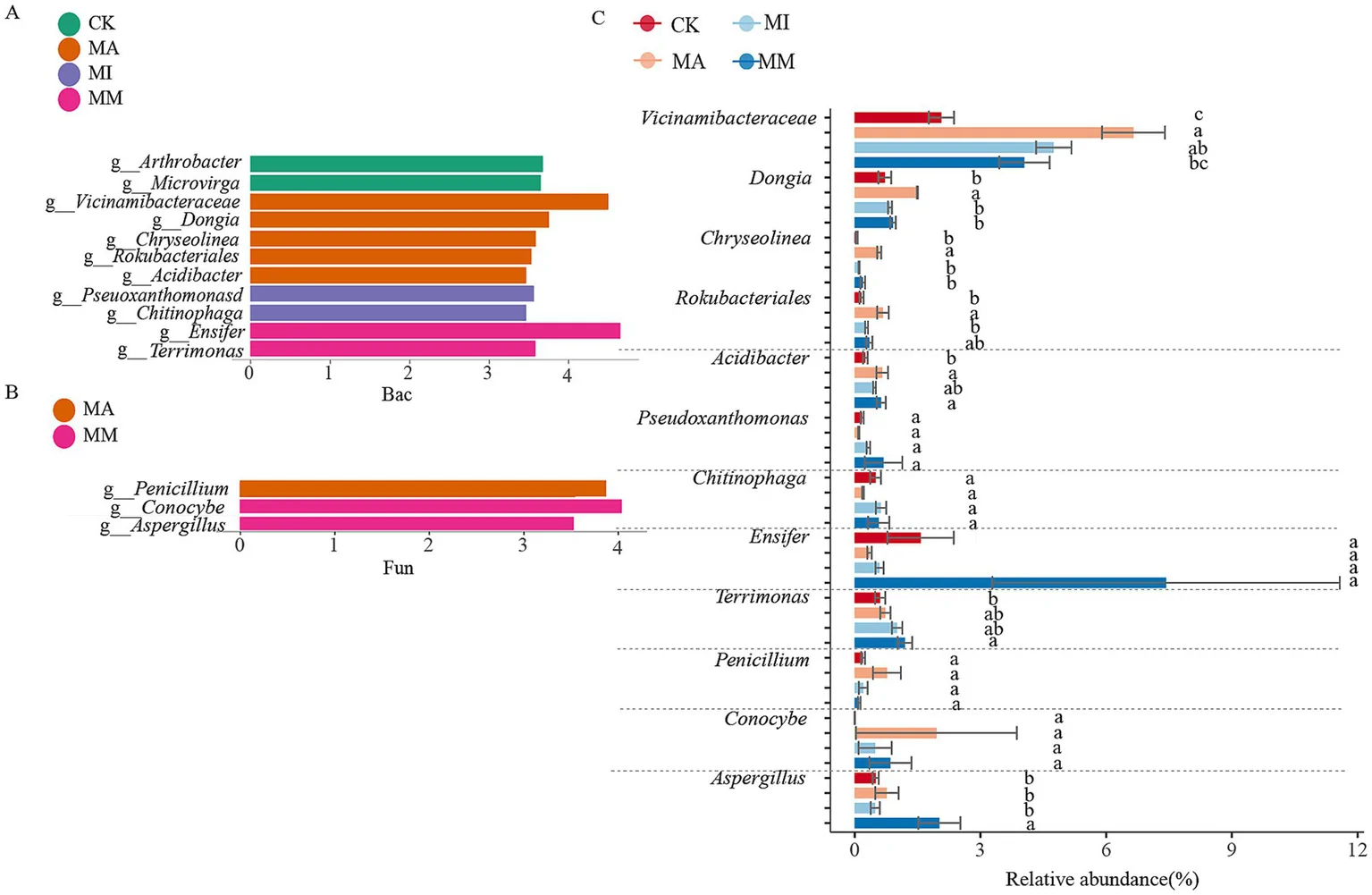
Differential bacterial and fungal taxa in rhizospheric soil identified by LEfSe analysis. **(A)** LEfSe-derived LDA scores of discriminative bacterial genera among the CK, MA, MI, and MM treatments in rhizospheric soil (LDA log score > 3.5, *p* < 0.05). **(B)** LEfSe-derived LDA scores of discriminative fungal genera across treatments using the same criteria. **(C)** Relative abundances of bacterial and fungal taxa identified as significantly enriched by LEfSe analysis, showing consistent abundance patterns across treatments.

To further validate these LEfSe results, we quantified the relative abundances of the differentially enriched bacterial and fungal taxa across treatments (Figure 4C). The abundance patterns were generally consistent with the LEfSe-based discrimination, and the enriched taxa all exhibited relative abundances exceeding 0.5%, indicating that these taxa represent ecologically meaningful members rather than rare community constituents.

### Effects of treatments on soybean growth and their microbial associations

We next examined whether these microbial differences were accompanied by corresponding changes in soybean growth and yield-related traits (Supplementary Table S3). Aboveground fresh weight was higher in the MA, MI, and MM treatments than in CK, with a significant increase observed under MM (Figure 5A). Similarly, underground fresh weight was consistently higher across all amended treatments, with MM showing the strongest effect (Figure 5B). Nodule number was significantly increased in the MM treatment compared with all other treatments (Figure 5C), while plant height was significantly greater in MA and MM than in CK (Figure 5D). Overall, the MM treatment exhibited the strongest positive effects on soybean growth-related traits and nodulation among all treatments.

**Figure 5:**
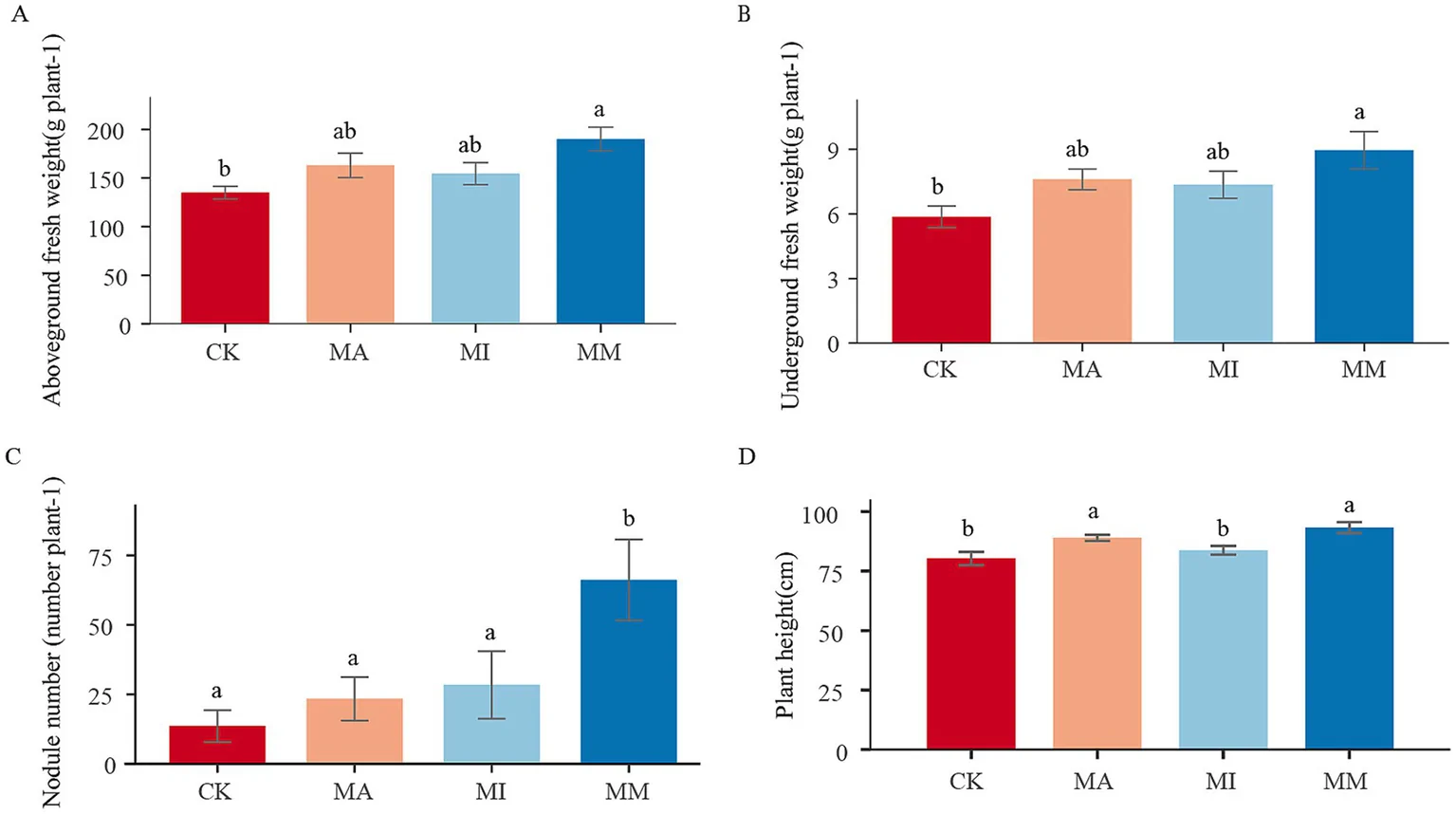
Effects of different soil amendments on soybean growth and nodulation traits. **(A)** Aboveground fresh weight, **(B)** underground fresh weight, **(C)** nodule number, and **(D)** plant height of soybean plants under the CK, MA, MI, and MM treatments. Bars represent means ± standard error. Different lowercase letters indicate significant differences among treatments based on one-way ANOVA followed by *post-hoc* tests (*p* < 0.05).

To investigate potential microbial taxa associated with these phenotypic variations, we conducted correlation analyses between rhizosphere microbial taxa and soybean growth-related traits. We first focused on nodule number, which exhibited the strongest treatment-dependent variation among all measured phenotypes. Microbial taxa stably present in soil (relative abundance > 0.5% for bacteria or > 0.3% for fungi in at least one treatment) were subjected to Spearman correlation analysis with nodule number. Several bacterial genera, including *Acidibacter*, *Bradyrhizobium*, *Ensifer*, and *Saccharimonadales*, showed significant positive correlations with nodule number (*p* < 0.05), while *Terrimonas* displayed a positive trend (*p* slightly > 0.05) (Figures 6A–E). In addition, four fungal genera—*Aspergillus*, *Conocybe*, *Fusidium*, and *Sarocladium*—were positively correlated with nodule number (Figures 6F–I). Notably, several of these taxa were also enriched in the MM treatment, consistent with its superior nodulation performance.

**Figure 6:**
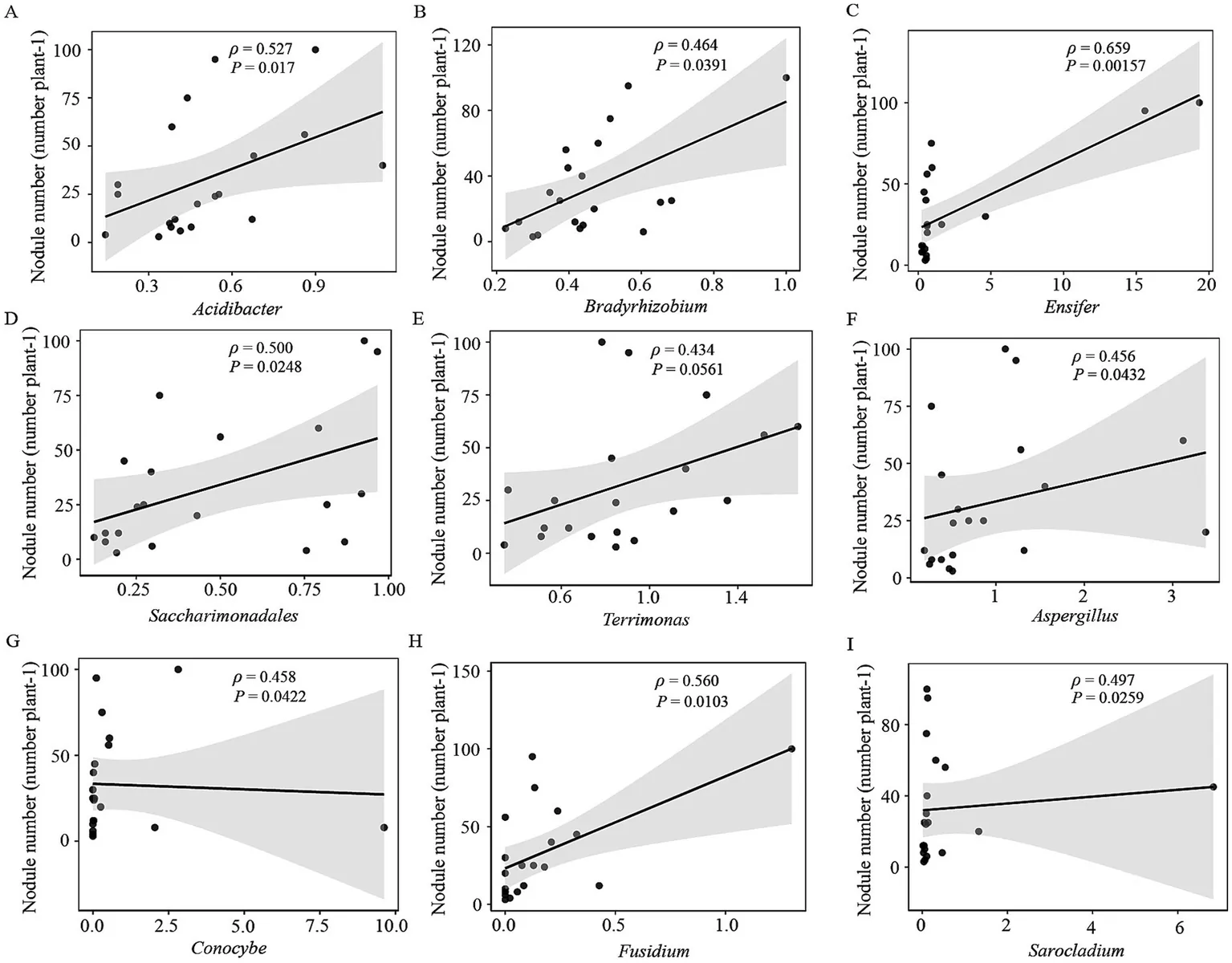
Correlations between rhizospheric microbial taxa and soybean nodule number. Spearman correlation analyses showing relationships between the relative abundances of selected bacterial genera **(A–E)** and fungal genera **(F–I)** and soybean nodule number across treatments (*p* < 0.05).

Given that the MM treatment harbored the highest number of nodulation-associated taxa, we further assessed whether these microbes were also linked to biomass accumulation. Additional Spearman correlation analyses revealed that *Terrimonas* and *Ensifer* were positively correlated with aboveground fresh weight, whereas *Ensifer* and the fungal genus *Conocybe* were positively associated with underground fresh weight (Figure 7). Collectively, these results suggest that a subset of microbes enriched in the MM treatment may simultaneously promote nodulation and biomass accumulation. Among them, *Ensifer* was consistently associated with nodule number as well as both aboveground and underground biomass, highlighting its potential role as a key multifunctional taxon linking rhizosphere microbial assembly with nitrogen fixation capacity and overall plant growth.

**Figure 7:**
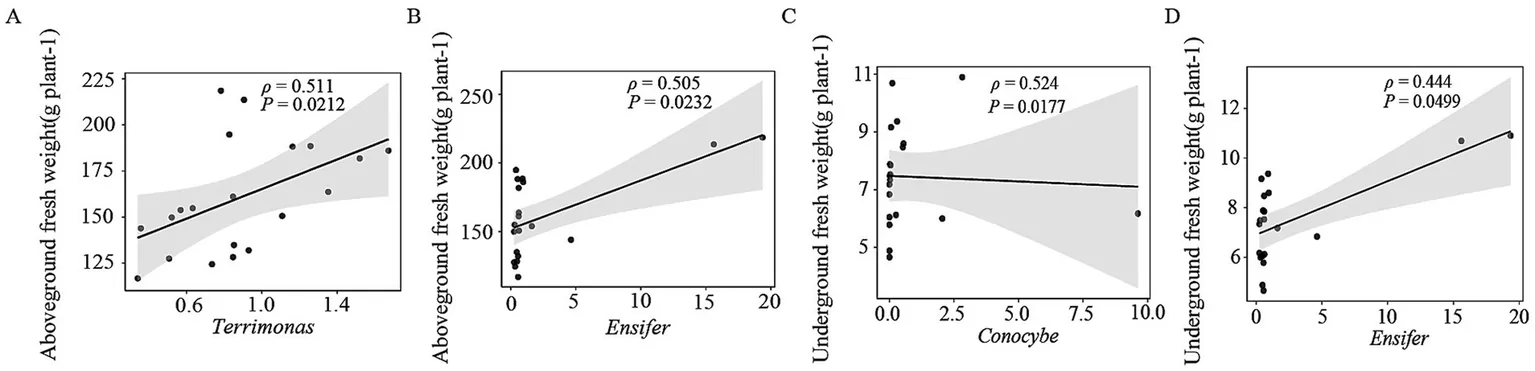
Correlations between nodulation-associated microbial taxa and soybean biomass traits. Spearman correlation analyses showing relationships between the relative abundances of selected bacterial and fungal genera and soybean aboveground fresh weight **(A,B)** and underground fresh weight **(C,D)** across treatments (*p* < 0.05).

## Discussion

The rhizosphere microbiome plays a pivotal role in crop productivity by regulating nutrient acquisition, plant growth, and stress resistance, and is therefore considered a key leverage point for sustainable agricultural management. Numerous studies have demonstrated that agricultural practices, including organic amendments and microbial inoculation strategies, can markedly influence soil microbial community composition and structure (Chen et al., 2025; Trabelsi and Mhamdi, 2013; Papin et al., 2024). However, the mechanisms by which plants regulate microbial assembly under different management practices remain unclear. In the present study, we show that different manure-based treatments exerted distinct effects on soil microbial communities, with treatment-induced shifts being consistently more pronounced in the rhizosphere than in bulk soil, and that these rhizospheric microbial changes were closely associated with soybean growth and nodulation (Figures 6, 7).

Across both bacterial and fungal communities, bulk soil generally exhibited weaker compositional differentiation among treatments compared with rhizospheric soil. Although certain phylum-level shifts were detectable in bulk soil, *β*-diversity analyses revealed limited separation among treatments, particularly for bacterial communities. In contrast, rhizospheric communities displayed clearer treatment-dependent clustering (Figure 1), especially under the MA and MM treatments. This pattern is consistent with the concept of a root-driven “filtration effect” (Dibbern et al., 2014), whereby plants act as ecological selectors that modulate microbial recruitment from the surrounding soil matrix (Park et al., 2023). Rhizosphere microbial assembly is widely described as a two-step process, in which microorganisms are first recruited from bulk soil to the root vicinity and subsequently filtered into the rhizosphere via plant-derived signals such as root exudates and cell-associated compounds (Turner et al., 2013; Bulgarelli et al., 2015; Edwards et al., 2015). In this context, the relatively modest variation in rhizospheric α-diversity observed here, despite clear compositional shifts, suggesting that treatments mainly altered community membership and relative abundance rather than overall richness. Such constrained diversity responses together with pronounced compositional restructuring highlight the dominant role of host-mediated selection in shaping rhizospheric microbiomes under different management regimes (Schmidt et al., 2019; Xu et al., 2024).

At the phylum level, rhizospheric bacterial communities were dominated by Proteobacteria and Acidobacteriota, consistent with previous reports from legume-associated soils (Chen et al., 2021). Actinobacteriota were consistently more abundant in rhizospheric soil than in bulk soil. Members of this phylum are well known producers of antimicrobial compounds (Bull et al., 2005), and may therefore contribute to pathogen suppression in the root zone. In contrast, Acidobacteriota declined in the rhizosphere, likely reflecting their preference for oligotrophic and more acidic environments (Jones et al., 2009; Kalam et al., 2020), whereas the rhizosphere is typically enriched in labile carbon substrates (Rosado-Porto et al., 2022). At finer taxonomic resolution, treatment-specific responses became more apparent (Pan et al., 2025; Li et al., 2023). The MA treatment increased *Vicinamibacteraceae* and *Acidibacter*, whereas the MM treatment selectively enriched *Ensifer* and *Terrimonas* (Figure 4). Among these, *Ensifer* is particularly notable. As a member of the Rhizobiales, *Ensifer* includes species capable of symbiotic nitrogen fixation and diverse plant-associated lifestyles (Geddes et al., 2021). Its enrichment under the MM treatment may reflect its ability to efficiently utilize root-derived carbon substrates (Lambert et al., 2001), facilitating competitive colonization of the root environment under combined manure–microbial amendment (Ollio et al., 2025). Importantly, the ecological relevance of *Ensifer* enrichment was further supported by its strong and consistent associations with soybean performance. *Ensifer* showed significant positive correlations with nodule number, aboveground biomass, and underground biomass (Figures 6, 7), suggesting a multifunctional role linking rhizosphere assembly with nitrogen acquisition and plant growth (Govindasamy et al., 2017). The selective enrichment of *Ensifer* under MM therefore provides a plausible microbial explanation for the enhanced nodulation and biomass accumulation observed in soybean.

Compared with bacteria, fungal communities showed generally weaker treatment separation in *β*-diversity (Supplementary Figure S3). However, MM significantly increased fungal richness, indicating that fungal communities responded to treatments primarily through richness and compositional changes rather than large-scale community restructuring (Finn et al., 2021). Ascomycota and Basidiomycota dominated fungal communities in both bulk and rhizospheric soils (Figure 3). The enrichment of Ascomycota in amended treatments, especially under MA, likely reflects their copiotrophic strategies and ability to rapidly utilize organic substrates introduced via manure application (Sun et al., 2023). Root exudates may further reinforce this pattern by providing readily available carbon substrates (Berendsen et al., 2012). At finer taxonomic levels, functional differentiation became evident. *Penicillium* was enriched under MA (Figure 4), consistent with its role in organic matter decomposition and nutrient mineralization (Prasanna et al., 2016). By comparison, the MM treatment increased *Conocybe* and *Aspergillus* (Figure 4), taxa associated with organic matter turnover and nutrient mobilization (Liu et al., 2023; Nascimento et al., 2021), which may indirectly facilitate plant nutrient acquisition. Notably, both genera were positively associated with nodulation, suggesting that fungal-mediated nutrient mobilization and organic matter transformation may help create a rhizosphere environment favorable for symbiotic nitrogen fixation and root development (Zhang et al., 2020; Singh et al., 2025).

Collectively, the convergence of treatment-induced microbial enrichment and phenotype-associated taxa suggests a functional linkage between rhizosphere microbial assembly and soybean performance (Moretti et al., 2024; Schmidt et al., 2019). In particular, the consistent enrichment and phenotype association of *Ensifer* across nodulation and biomass traits highlight its potential role as a multifunctional keystone taxon linking nitrogen acquisition capacity with plant growth (Shakoor et al., 2023; Wang et al., 2024). These results suggest that management drives rhizosphere microbiomes toward functionally optimized communities that enhance plant performance. Although causal relationships remain to be validated, the integration of community profiling, differential abundance analysis, and phenotype association provides a mechanistic basis linking rhizosphere microbial assembly with plant productivity. Future studies integrating stable isotope tracing (Wei and Huang, 2025), meta-omics approaches (Clagnan et al., 2024), and isolate-based functional assays (Sarhan et al., 2019) will be essential for confirming the functional roles of candidate taxa such as *Ensifer* and for translating rhizosphere microbiome insights into practical agronomic applications.

## Conclusion

This study demonstrated that soil amendments, particularly the combined application of manure and microbial inoculants (MM), promoted soybean growth and root nodulation under field conditions in a wheat-soybean rotation system. Among all tested treatments, MM produced the greatest improvement in soybean growth, as indicated by increased aboveground and belowground biomass, greater plant height, and enhanced nodule formation compared with single-amendment treatments and the untreated control. The superior performance of MM was accompanied by distinct changes in soybean rhizosphere microbial communities, including increased fungal richness and the selective enrichment of plant-associated taxa, particularly the nitrogen fixation-associated genus *Ensifer*. The positive associations between MM-enriched microbial taxa and soybean nodulation and biomass accumulation suggest that these taxa may contribute to the growth-promoting effects of combined amendment. Collectively, our findings indicate that the combined application of organic manure and microbial inoculants was more effective than either amendment alone in promoting soybean growth and root nodulation, potentially through the recruitment of beneficial rhizosphere microorganisms. These findings should be interpreted in the context of the specific field conditions and soybean growth stage investigated. Future studies across multiple environments and seasons, combined with functional validation approaches, will further clarify the underlying mechanisms and broader applicability of microbiome-mediated plant growth promotion.

## Data Availability

The datasets presented in this study can be found in online repositories. The names of the repository/repositories and accession number(s) can be found in the article/Supplementary material.
