# Supplementary material for: Combined application of organic and microbial amendments reshapes soil microbiome diversity and improves soybean growth performance
Source: Front Microbiol. 2026 Sep 18;17:1939259. doi: 10.3389/fmicb.2026.1939259 (PMC13630738; doi:10.3389/fmicb.2026.1939259)
Supplement: Supplementary file 1 [file Supplementary_file_1.pdf]

Fig. S1.

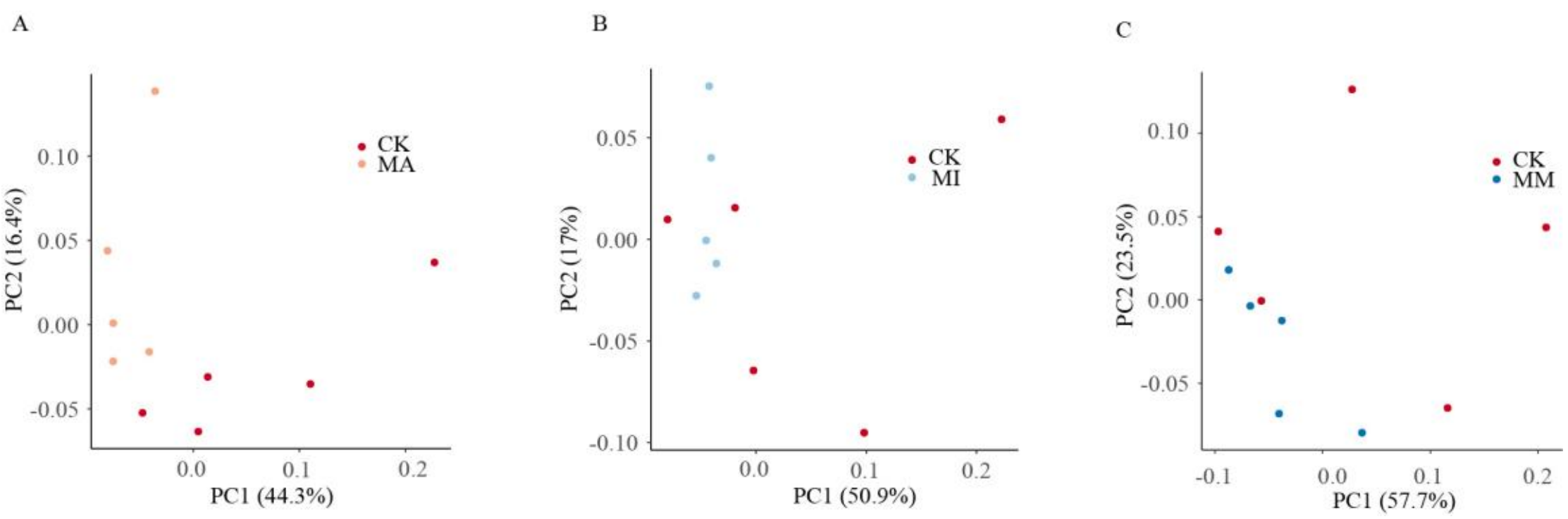
A
B
C
CK
MA
MI
MM
PC1 (44.3%)
PC2 (16.4%)
PC1 (50.9%)
PC2 (17%)
PC1 (57.7%)
PC2 (23.5%)

Fig. S2.

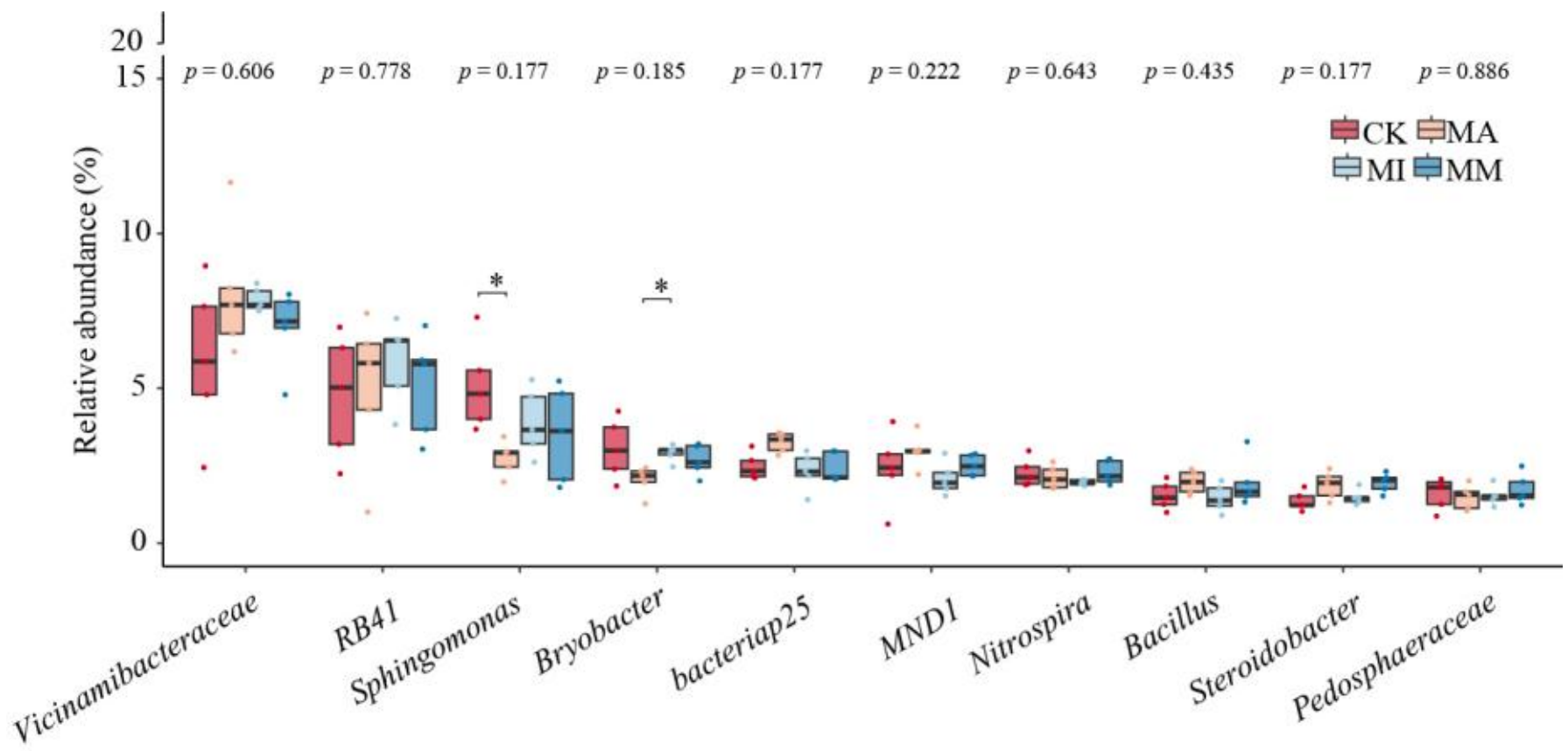
p = 0.606
p = 0.778
p = 0.177
p = 0.185
p = 0.177
p = 0.222
p = 0.643
p = 0.435
p = 0.177
p = 0.886
CK
MA
MI
MM
Relative abundance (%)
Vicinamibacteraceae
RB41
Sphingomonas
Bryobacter
bacteriap25
MND1
Nitrospira
Bacillus
Steroidobacter
Pedosphaeraceae

Fig. S3.

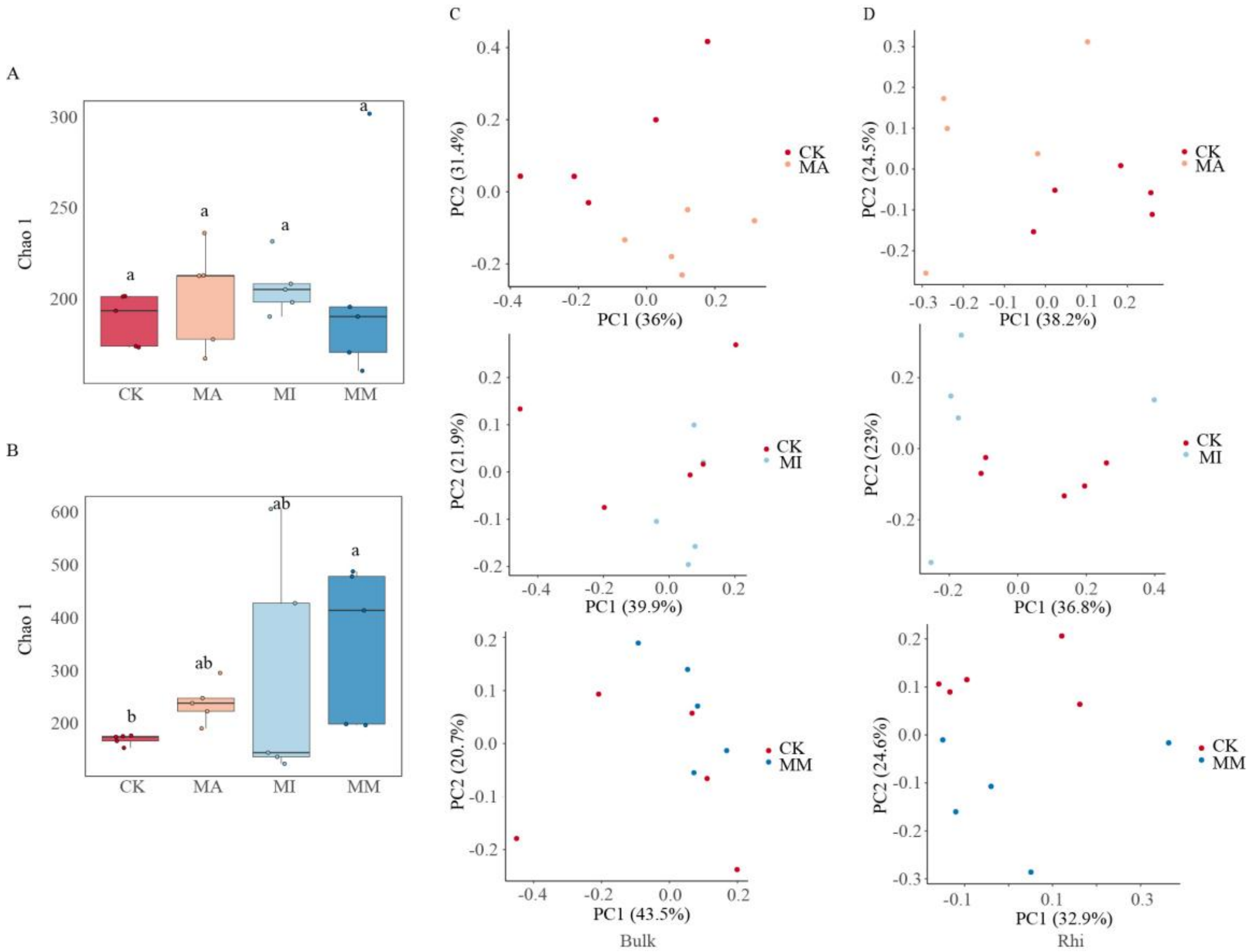

A
B
C
D
Chao 1
CK
MA
MI
MM
PC1 (36%)
PC2 (31.4%)
PC1 (38.2%)
PC2 (24.5%)
PC1 (39.9%)
PC2 (21.9%)
PC1 (36.8%)
PC2 (23%)
PC1 (43.5%)
PC2 (20.7%)
PC1 (32.9%)
PC2 (24.6%)
Bulk
Rhi

Fig. S4.

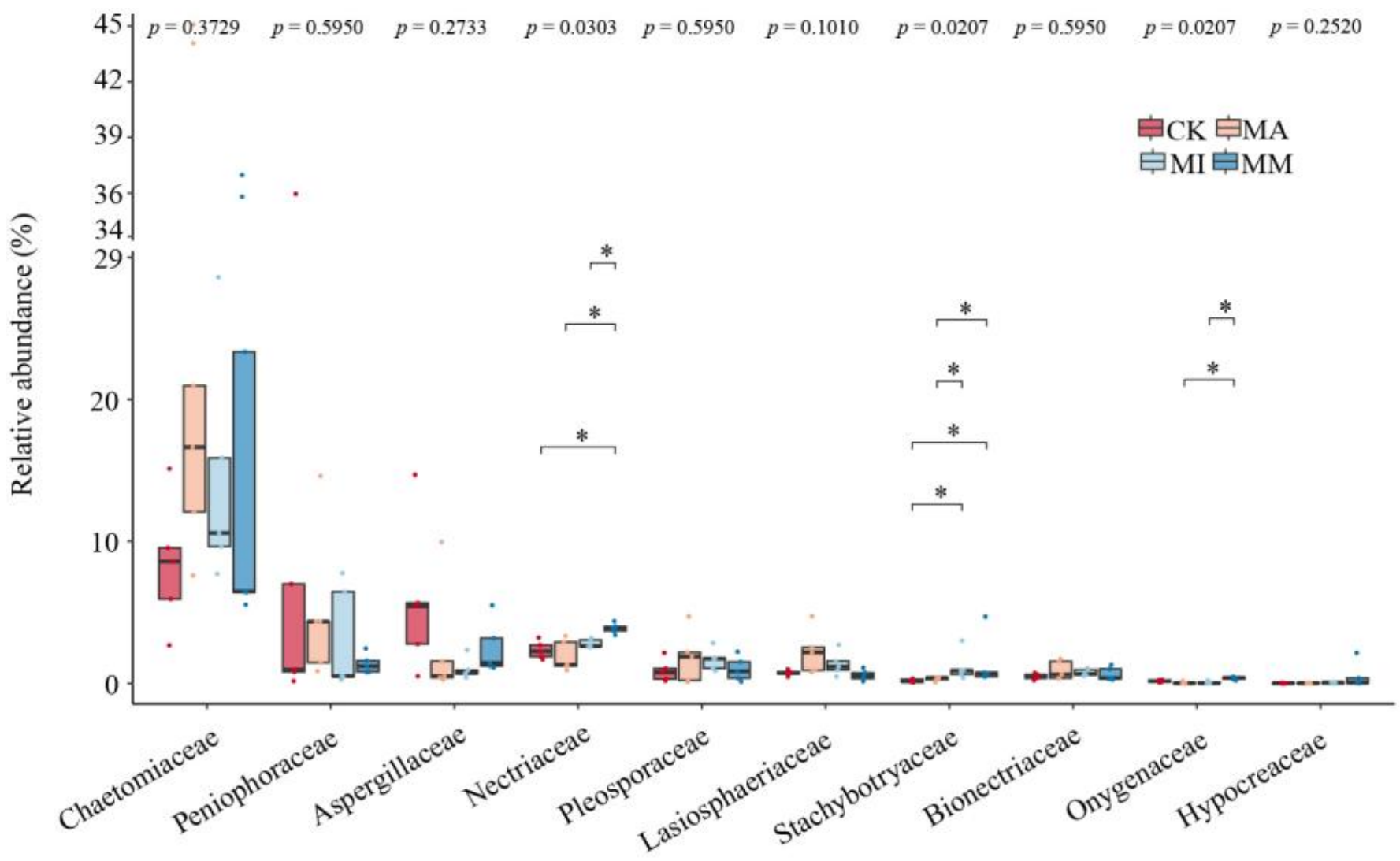

p = 0.3729
p = 0.5950
p = 0.2733
p = 0.0303
p = 0.5950
p = 0.1010
p = 0.0207
p = 0.5950
p = 0.0207
p = 0.2520
CK
MA
MI
MM
Relative abundance (%)
Chaetomiaceae
Peniophoraceae
Aspergillaceae
Nectriaceae
Pleosporaceae
Lasiosphaeriaceae
Stachybotryaceae
Bionectriaceae
Onygenaceae
Hypocreaceae

Fig. S1. (A-C) Principal coordinate analysis (PCoA) of bulk bacterial community composition based on weighted UniFrac distances, showing pairwise comparisons between each treatment and the control (CK): MA vs. CK (A; PC1 = 44.3%, PC2 = 16.2%), MI vs. CK (B; PC1 = 50.9%, PC2 = 17%), and MM vs. CK (C; PC1 = 57.7%, PC2 = 23.5%). Treatments include untreated control (CK), cattle manure amendment (MA), commercial microbial inoculant (MI), and combined manure-based inoculant treatment (MM).

Fig. S2. Relative abundances of the top 10 most abundant bacterial genera in bulk soil across treatments.

Fig. S3. Alpha diversity and beta diversity of fungal communities under different field treatments. (A, B) Chao1 α-diversity of fungal communities in bulk soil (A) and rhizospheric soil (B). Different lowercase letters indicate significant differences among treatments based on one-way ANOVA followed by LSD post hoc tests ($P < 0.05$). (C, D) Principal coordinate analysis (PCoA) of bulk and rhizospheric fungal community composition based on weighted UniFrac distances, showing pairwise comparisons between treatments and the control. Treatments include the untreated control (CK), cattle manure amendment (MA), commercial microbial inoculant (MI), and the combined manure-based inoculant treatment (MM).

Fig. S4. Relative abundance of the top 10 most abundant fungal families in bulk soil across treatments.

Supplementary Table S1.

A. Bacteral PCR amplification program.

Temperature (°C)	Time	Cycles
94℃	5min	1
94℃	30s	30
53℃	30s	30
72℃	30s	30
72℃	8min	1
12℃	∞	1

B. PCR amplification system.

Component	Volume
Taq enzyme	25 μL
H2O	Up to 50 total volume
Forward primer（10μm）	2.5 μL
Reverse primer（10μm）	2.5 μL
DNA	~50 ng
BSA	/

Supplementary Table S2.

A. Fungal PCR amplification program.

Temperature (°C)	Time	Cycles
95℃	3min	1
95℃	20s	33
56℃	20s	33
72℃	30s	33
72℃	5min	1
12℃	∞	1

B. PCR amplification system.

Component	Volume
Taq enzyme	25 μL
H2O	Up to 50 total volume
Forward primer（10μm）	2 μL
Reverse primer（10μm）	2 μL
DNA	~50 ng
/	/

Supplementary Table S3.

Aboveground fresh weight (g $plant^{-1}$)

CK	MA	MI	MM
153.832	194.769	161.1	181.823
143.924	154.808	134.89	186.049
128.295	188.171	188.519	218.538
116.766	149.813	124.421	150.631
131.984	127.511	163.657	213.55

Underground fresh weight (g $plant^{-1}$)

CK	MA	MI	MM
7.18	9.16	7.527	8.472
6.832	7.479	6.05	8.594
4.88	7.879	9.366	10.9
4.66	7.339	6	6.121
5.779	6.17	7.845	10.688

Nodule number (number $plant^{-1}$)

CK	MA	MI	MM
25	45	24	56
30	12	10	60
3	40	75	100
4	12	8	20
6	8	25	95

Plant height (cm)

CK	MA	MI	MM
68.35	93.39	87.65	92.75
96.71	88.89	87.02	98.95
83.39	90.21	87.36	92.84
83.85	95.21	83.05	89.39
79.78	88.32	89.75	96.94
88.15	91.39	77.2	103.36
82.53	89.91	79.99	96.68
74.61	85.79	71.99	94.74
78.49	85.38	84.25	90.62
67.31	81.43	89.11	76.25
